# Robotic left hepatectomy for the management of mucinous cystic neoplasm of the liver with atypical presentation inside the biliary tract: a case report

**DOI:** 10.1093/jscr/rjaf339

**Published:** 2025-05-27

**Authors:** Santiago Andrés Muñoz-Palomeque, Miguel Dávila Quiroz, Geylor Acosta, Ligia M Redroban, Martha Cueva, Frans Ivan Serpa

**Affiliations:** General and Laparoscopic Surgery, International University of Ecuador, Metropolitan Hospital of Quito, Quito 170521, Ecuador; General and Laparoscopic Surgery, International University of Ecuador, Metropolitan Hospital of Quito, Quito 170521, Ecuador; Videolaparoscopy and Robotics Surgery, Hospital Metropolitano, Quito 170521, Ecuador; Pathology Service, Metropolitan Hospital, Quito 170521, Ecuador; Videolaparoscopy and Robotics Surgery, Hospital Metropolitano, Quito 170521, Ecuador; Hepatobiliopancreatic, Videolaparoscopy and Robotics Surgery, Hospital Metropolitano, Quito 170521, Ecuador

**Keywords:** biliary tract neoplasms, common bile duct, hepatectomy, mucinous cystic neoplasm of the liver, ovarian-type subepithelial stroma, robotic surgical procedures

## Abstract

Mucinous cystic neoplasm of the liver (MCN-L) is a rare epithelial tumor with ovarian-like stroma (OLS), accounting for less than 5% of hepatic cysts. While usually confined to the liver, MCN-L rarely invades the biliary tract, complicating diagnosis and treatment. This case report describes a 53-year-old woman with recurrent cholangitis after robotic hepatic resection for MCN-L. Imaging revealed a lesion in the bile ducts, initially suspected to be intraductal papillary neoplasm of the bile duct (IPNB). Robotic left hepatectomy using the Da Vinci platform was performed, and intraoperative findings confirmed an intraductal pedunculated mass. Histopathology identified MCN-L with OLS invading the biliary tract. The patient recovered uneventfully and was discharged on postoperative day two. Although rare, MCN-L with biliary invasion can mimic IPNB. Robotic surgery provides a minimally invasive solution, emphasizing the importance of early detection and intervention for optimal outcomes.

## Introduction

Cystic hepatobiliary neoplasms with mucin-producing epithelium include mucinous cystic neoplasm of the liver (MCN-L) and intraductal papillary neoplasm of the bile duct (IPNB). These are rare and distinct entities with unique clinical, pathological, and imaging features. The defining feature of MCN-L is the presence of subepithelial ovarian-like stroma (OLS), which differentiates it from IPNB [1, 2].

MCN-L, previously referred to as cystadenomas or cystadenocarcinomas, is a rare epithelial tumor that arises from the liver parenchyma or, less frequently, in the extrahepatic bile ducts. It predominantly affects women, with an age range of 28–76 years, and has an incidence of 1 case per 200 000–1 million people annually. MCN-L accounts for less than 5% of liver cysts, with fewer than 250 cases reported globally as of 2021 [3–7].

This case report describes a rare presentation of MCN-L with biliary duct invasion, managed successfully with robotic left hepatectomy.

## Case report

A 53-year-old female with a history of robotic hepatic resection for a mucinous cyst 1-year prior presented with recurrent cholangitis over the past 6 months. Symptoms included right upper quadrant abdominal pain, jaundice, and fever, which resolved with antibiotics and endoscopic retrograde cholangiopancreatography (ERCP).

**Figure 1:**
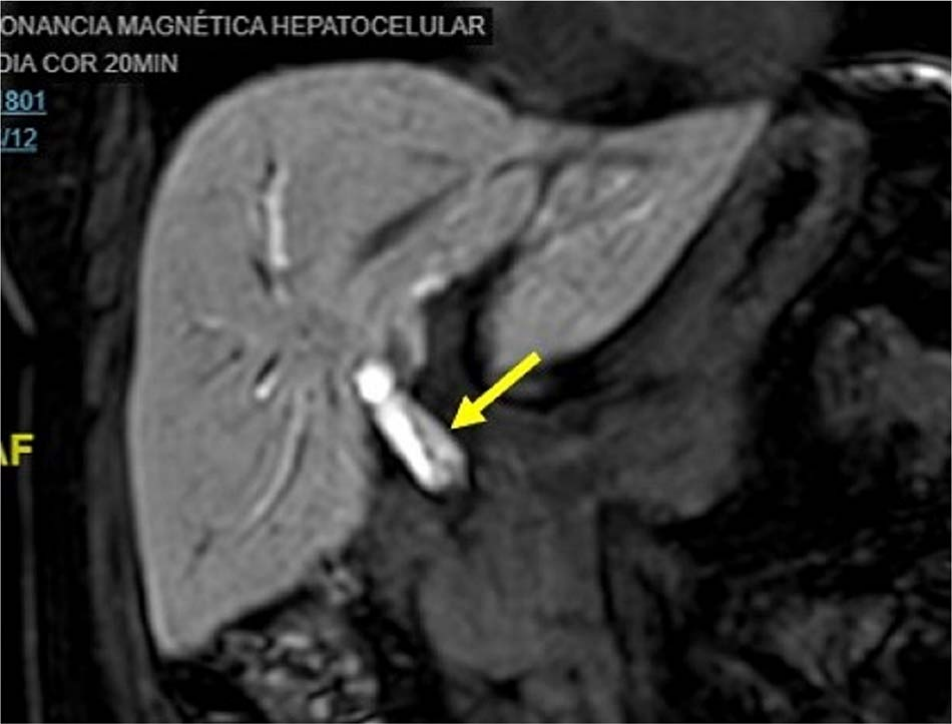
Magnetic resonance cholangiopancreatography image common bile duct intraductal tumor, coronal view.

**Figure 2:**
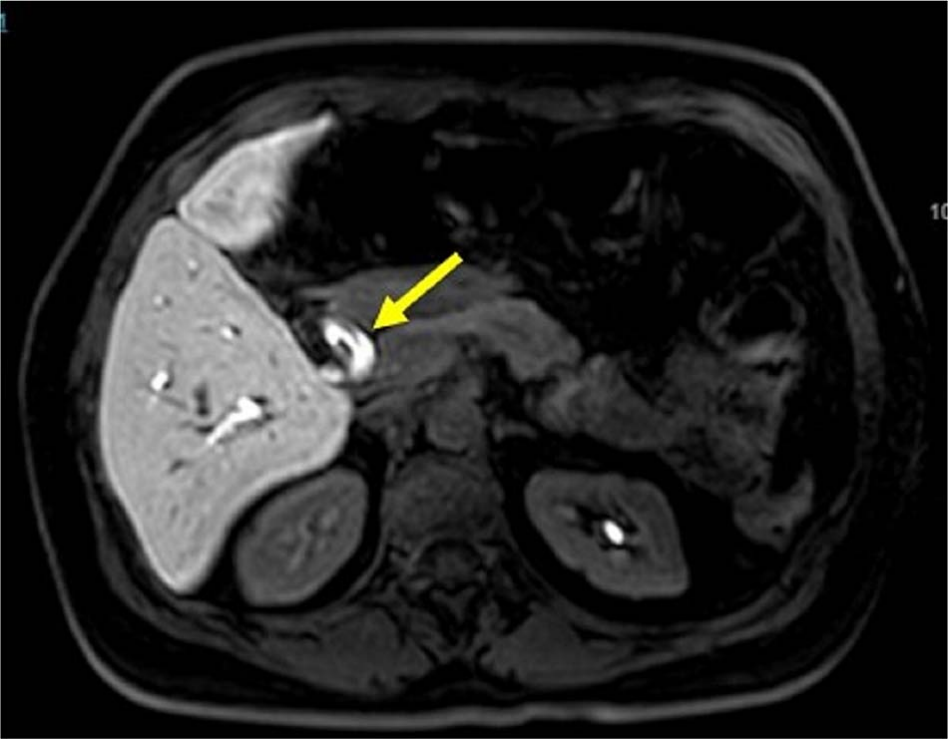
Magnetic resonance cholangiopancreatography image common bile duct intraductal tumor, axial view.

Initial imaging with ultrasound and computed tomography (CT) revealed intrahepatic bile duct dilatation without evidence of stones. Magnetic resonance cholangiopancreatography (Figs 1 and 2) showed a solid intraductal lesion extending from the left bile duct to the common bile duct, raising suspicion for IPNB versus MCN-L. SpyGlass endoscopy confirmed the presence of a solid intraductal tumor. Laboratory findings, including bilirubin, alpha-fetoprotein, and carcinoembryonic antigen, were within normal limits.

Given the high suspicion for IPNB or MCN-L, surgical resection was planned. Robotic left hepatectomy was chosen as the surgical approach after obtaining informed consent from the patient.

Intraoperatively, the Da Vinci robotic platform was utilized. Adhesions from previous surgeries were released using a robotic vessel sealer. The hepatic hilum was identified, and the left hepatic artery and portal branch were isolated and transected for ischemic demarcation. Indocyanine green was administered to delineate the bile ducts. A pedunculated intraductal mass was identified within the left hepatic duct, which was transected. A frozen section of the proximal duct confirmed negative margins.

**Figure 3:**
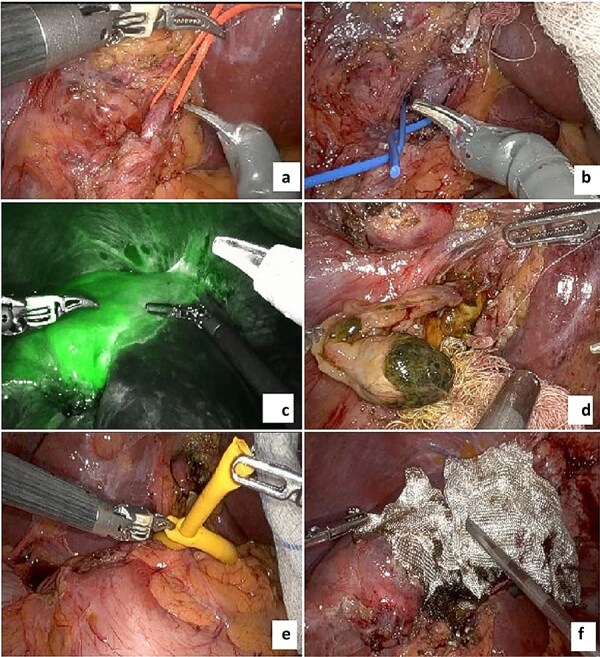
Images of the procedure. (a) Left hepatic artery; (b) left portal vein; (c) indocyanine green ischemic demarcation before liver transection; (d) intraductal pedunculated solid mass; (e) Pringle maneuver using Foley catheter; (f) final transection with hemostatic material.

The Pringle maneuver was prepared using a Foley catheter, and parenchymal transection was performed using a mechanical linear stapler, bipolar energy, and a vessel sealer. The specimen was retrieved and sent for pathology. Estimated blood loss was 300 milliliters (Fig. 3). Postoperatively, the patient recovered without complications, resuming her diet and having her drain removed by postoperative day two.

**Figure 4:**
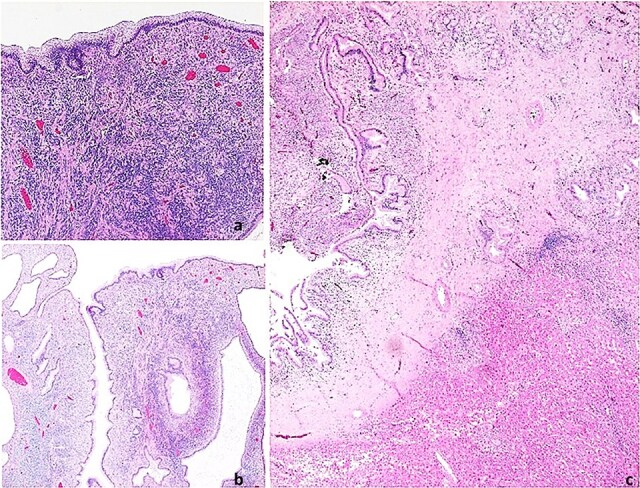
Histopathological images. Mucinous cystic neoplasm of the liver. (a) Neoplasm with a thick fibrous pedicle anchored in the hepatic parenchyma; (b) Multilocular neoplasm with compact cellular stroma; (c) Mucin-producing columnar epithelial lining and underlying ovarian-type hypercellular stroma.

Pathological analysis confirmed MCN-L with biliary tract invasion. The tumor measured 1 centimeter with a pedicle extending over 5 centimeters into the bile duct. It was characterized by microcystic structures lined by mucin-producing columnar epithelium with basal nuclei, no atypia, and ovarian-like stroma under the epithelium (Fig. 4).

## Discussion

MCN-L is a rare epithelial tumor of the liver, predominantly occurring in women. Its etiology is unclear, but its female predominance, age of onset, and hormonally responsive OLS suggest ectopic organogenesis during embryonic development [8]. Histologically, MCN-L is defined by a cystic structure lined with mucinous epithelium and OLS beneath it [5].

The differential diagnosis includes IPNB, intrahepatic cholangiocarcinoma with cystic changes, echinococcal cysts, and simple cysts [3]. Unlike MCN-L, IPNB often communicates with the bile ducts and lacks OLS. IPNB is also associated with older age and invasive carcinoma [1, 7, 9]. While MCN-L typically does not involve the bile ducts, Kozaka *et al*. [10] reported biliary prolapse in 15% of cases.

MCN-L with biliary involvement is particularly rare and poses diagnostic challenges. Patients may present early due to obstructive jaundice or cholangitis [11]; however, they are generally considered benign and indolent hepatic cystic lesions, with no significant clinical symptoms in most patients, which generates difficulties in presurgical differential diagnosis [3, 6, 12]. Imaging plays a key role in characterizing hepatic cystic lesions. Ultrasound, CT, and magnetic resonance imaging (MRI) are essential, with MRI being particularly useful for detecting septations and cyst-in-cyst appearances [13, 14]. SpyGlass and other advanced endoscopic techniques can provide additional diagnostic clarity.

Histopathology remains the gold standard for diagnosis. The hallmark feature of OLS is pathognomonic for MCN-L [2, 8]. Xiao *et al*. [15] identified four key imaging variables for diagnosing MCN-L: septation that arises within a wall without indentation, multiple septa, intracapsular cyst signs, and solitary lesions. Despite advances in imaging and diagnostic guidelines, preoperative identification remains challenging [13].

Surgical resection is the treatment of choice for MCN-L, allowing definitive diagnosis and preventing malignant transformation [3, 4, 14]. While MCN-L is typically benign, it is considered a premalignant lesion capable of invasive behavior [6, 8]. Complete resection ensures favorable outcomes, as demonstrated in this case.

The prognosis is excellent, with good survival after complete resection in case of correct initial diagnosis; however, relapses have also been reported, reinforcing the need for early surgical intervention [3, 5, 6, 14].

Robotic surgery offers advantages over traditional approaches, including enhanced visualization, precision, and reduced morbidity. This case demonstrates the efficacy of robotic hepatectomy in achieving R0 resection. Similar favorable outcomes have been reported in the few documented cases of biliary-invasive MCN-L.

Biliary invasion in MCN-L is exceedingly rare. Srinivas *et al*. [11] reported a case of a 31-year-old female with obstructive jaundice and cholangitic abscess caused by MCN-L involving the bile ducts. Left hemihepatectomy revealed a multiloculated cystic lesion with OLS. Similarly, Fukui *et al*. [9] described a 69-year-old woman with obstructive jaundice and a cystic lesion protruding into the bile ducts. Surgical resection confirmed MCN-L with OLS. These cases underscore the importance of histopathology in confirming the diagnosis and highlight the challenges in differentiating MCN-L from IPNB preoperatively.

## Conclusion

MCN-L is a rare cystic hepatic neoplasm with an incidence of less than 5% among liver cysts. It typically presents asymptomatically, leading to delayed diagnoses. Biliary invasion is an extremely rare manifestation, often misdiagnosed as IPNB. The presence of OLS is critical for definitive diagnosis.

Surgical resection remains the cornerstone of management, allowing both diagnosis and curative treatment. Robotic approaches, as demonstrated in this case, offer a minimally invasive alternative with excellent outcomes. Early identification and management are key to preventing complications and ensuring favorable prognoses in these patients.
